# Incorporating Water Molecules into Highly Accurate Binding Affinity Prediction for Proteins and Ligands

**DOI:** 10.3390/ijms252312676

**Published:** 2024-11-26

**Authors:** Diya Zhang, Qiaozhen Meng, Fei Guo

**Affiliations:** 1 School of Computer Science and Engineering, Central South University, Changsha 410000, China; diya.zhang@csu.edu.cn; 2 School of Computer Science, Xiangtan University, Xiangtan 411105, China

**Keywords:** water molecules, protein, ligand, binding affinity, GNN

## Abstract

In the binding process between proteins and ligand molecules, water molecules play a pivotal role by forming hydrogen bonds that enable proteins and ligand molecules to bind more strongly. However, current methodologies for predicting binding affinity overlook the importance of water molecules. Therefore, we developed a model called GraphWater-Net, specifically designed for predicting protein–ligand binding affinity, by incorporating water molecules. GraphWater-Net employs topological structures to represent protein atoms, ligand atoms and water molecules, and their interactions. Leveraging the Graphormer network, the model extracts interaction features between nodes within the topology, alongside the interaction features of edges and nodes. Subsequently, it generates embeddings with attention weights, inputs them into a Softmax function for regression prediction, and ultimately outputs the predicted binding affinity value. Experimental results on the Comparative Assessment of Scoring Functions (CASF) 2016 test set show that the introduction of water molecules into the complex significantly improves the prediction performance of the proposed model for protein and ligand binding affinity. Specifically, the Pearson correlation coefficient (*R_p_*) exceeds that of current state-of-the-art methods by a margin of 0.022 to 0.129. By integrating water molecules, GraphWater-Net has the potential to facilitate the rational design of protein–ligand interactions and aid in drug discovery.

## 1. Introduction

Exploring the binding affinity between proteins and ligands is an important topic in computer-aided drug design (CADD) [1,2,3], as it is essential for researching and developing new drugs [4,5,6,7,8]. Structure-based drug design (SBDD) is a key method for achieving CADD [9]. Binding affinity is typically quantified by the equilibrium dissociation constant *K_d_*, which indicates the strength of the interaction between a ligand and its protein target. Additional related metrics include the inhibition constant *K_i_*, which measures the potency of an inhibitor binding to a protein, and the half maximal inhibitory concentration *IC*_50_, which denotes the concentration of a drug or inhibitor required to achieve 50% inhibition of a specific biological process [4,6,10,11,12,13].

At present, methods for calculating or estimating binding affinity are generally categorized based on their computational approach and accuracy, including end-point free energy methods, rigorous free energy calculation methods, machine learning (ML) methods and scoring functions. End-point free energy methods utilize molecular dynamics (MD) simulation trajectories, integrating molecular mechanics with solvation models to estimate binding free energy [14]. Notably, two widely adopted approaches within this category are molecular mechanics Poisson–Boltzmann surface area (MM/PBSA) and molecular mechanics generalized born surface area (MM/GBSA) [15]. Both methods offer a pragmatic balance between accuracy and computational cost. Conversely, rigorous free energy calculation methods are characterized by their high accuracy, albeit at the expense of significant computational resources. These methods thoroughly account for detailed thermodynamic properties and necessitate extensive MD simulations [16]. Key techniques in this realm include free energy perturbation (FEP), thermodynamic integration (TI), and steered molecular dynamics (SMD), which often employ either alchemical or physical pathway-based approaches to estimate free energy differences between states. Additionally, ML methods trained on large datasets can efficiently predict binding free energies, thus serving as valuable tools in drug discovery [17]. However, the accuracy of these methods is contingent upon the quality of the training data. They may encounter challenges in capturing complex physicochemical interactions, including solvent effects, entropy, and generalizing to novel compounds when the training data are limited.

Scoring functions are estimation methods used to quickly assess the binding affinity in molecular docking. These include physics-based, empirical, knowledge-based, and descriptor/ML-based scoring functions [2,6,18,19,20,21]. Physics-based scoring functions use physical principles and force fields to provide rapid binding affinity estimates [19,22]. Yin et al. proposed the MedusaScore scoring function, which evaluates protein–ligand binding by incorporating van der Waals attraction and repulsion, solvation energy, and hydrogen bonding energies—calculated between atomic backbones and side chains, and between backbones and side chains. The predicted binding affinity of complexes in the PDBbind (version 2005) dataset was achieved with a correlation coefficient of 0.61 with experimental affinity [23]. Empirical scoring functions, derived from fitting computational terms to experimental data, combine various energy components with weighted coefficients to predict binding affinity [24]. The LISA scoring function, developed by Zheng et al., utilizes interaction descriptors to improve the prediction accuracy [25]. Knowledge-based scoring functions leverage data from known experimental structures to predict binding affinities [22,26]. For example, DrugScore by Gohlke et al. [27] transformed 3D structural information into pairwise interaction potentials. Descriptor/ML-based scoring functions use structural descriptors or features derived from machine learning algorithms to predict binding affinity [3]. For instance, Ballester et al. developed a scoring function based on random forests, considering the nine most commonly occurring element types in proteins and ligands [28].

Both proteins and ligands exist in a solvated water environment before binding. The binding of proteins and ligands involves desolvation, which requires energy input to disrupt the interactions between water molecules and the protein or ligand. Whether the overall binding process is energetically favorable depends on the balance between desolvation, new interactions formed, and solvent entropy changes. Therefore, the driving force behind protein–ligand binding is the change in binding free energy ∆G, calculated according to the following equation:

(1) ∆G=∆H−T∆S

∆H is the enthalpy change, while T∆S denotes the entropy change. If ∆G<0, the binding process occurs spontaneously. If T∆S<0, the entropy change is unfavorable and favorable ∆H is required to counterbalance it. Likewise, if ∆H is unfavorable and ∆H>0, favorable entropy compensation (i.e., T∆S>0) is needed to facilitate binding [29]. During protein–ligand binding, water molecules interact with proteins and ligands, impacting entropy and enthalpy changes, thereby affecting ∆G [30]. Water molecules can affect binding free energy through various mechanisms, such as water displacement from binding sites, alterations in hydrogen bonding, solvent reorganization, and hydrophobic effects [31]. For water displacement, when a ligand binds to a protein, water molecules initially occupying the binding site are often displaced into the bulk solvent. This release of ordered water molecules into the surrounding solvent increases the T∆S of the system, as the released water molecules become more disordered in bulk solvent. Such an increase in entropy is typically favorable and lowers the free energy (∆G), promoting a more spontaneous binding process. Hydrogen bonding changes can also play a significant role. When the protein–ligand interaction replaces hydrogen bonds that water molecules previously formed with the protein and ligand, it can affect the system’s enthalpy. If stronger hydrogen bonds are created between the protein and ligand, ∆H decreases, thus stabilizing the complex. Conversely, if hydrogen bonds are broken without equivalent replacements, the enthalpy may increase, making the binding less favorable. In some cases, water molecules can mediate hydrogen bonds between the protein and ligand, forming indirect, water-mediated hydrogen bonds. These interactions can stabilize the complex and reduce the enthalpy (∆H), though they may not be as favorable as direct protein–ligand hydrogen bonds. The balance between the disruption of hydrogen bonds among water, protein, and ligand and the formation of new hydrogen bonds in the protein–ligand complex, including water-mediated interaction, can significantly influence the ∆H changes during binding. Favorable hydrogen bonding interactions reduce ∆H and contribute to more favorable binding [32]. In summary, water displacement generally contributes to favorable entropy changes (T∆S>0), while hydrogen bonding changes can impact enthalpy (∆H) either positively or negatively, both of which ultimately impact the overall ∆G. The Gibbs Free Energy equation is as follows:

(2) $$∆G=RTlnK_{d}$$

In Equation (2), *R* is the ideal gas constant and *T* is the absolute temperature. A logarithmic relationship exists between ∆G and binding affinity, where at a certain temperature, a more negative ∆G is associated with a smaller *K_d_*, indicating a stronger binding affinity between proteins and ligands. The presence of water molecules affects the binding affinity between ligands and their target molecules by influencing both enthalpy and entropy, which in turn impacts ∆G [5]. Since ∆G is related to $K_{d}$ through Equation (2), it is crucial to account for the influence of water molecules—such as through water displacement and changes in hydrogen bonding—when calculating protein–ligand affinity [33].

The methods previously mentioned do not consider the influence of water molecules on binding affinity. Therefore, this study aims to investigate how water molecules at the protein–ligand interface affect binding affinity. Firstly, two methods were used to predict the sites of water molecules, forming a water network integrated with the complex structures in the PDBbind dataset. Then, the atoms, covalent bonds, and interactions were transformed into graph structures, and node and edge features were extracted. These two obtained graphs were input into the Graphormer network [34] for feature extraction, with binding affinity predicted via the Softmax regression function [35] after connecting the two graphs. Simultaneously, we predicted the binding affinity of complexes without a water network to explore the role of water molecules. Experimental results have shown that the introduction of water molecules into the binding pocket significantly improves the *R_p_* value, and the proposed method, GraphWater-Net, achieves the best performance on CASF-2016 [21] compared to other advanced methods for predicting binding affinity.

## 2. Results and Discussion

### 2.1. Overview

Firstly, we employed two established methods for predicting water molecule sites to identify hydration sites in the binding pocket and pinpoint key water molecules involved in the binding process. The proposed method integrates the predicted water molecule information into the complex, converting it into a graph structure. The complex graph with a water network was input into a feature extraction network for feature extraction, and finally, regression prediction of binding affinity was performed. Information about the dataset can be found in Section 3.1.

### 2.2. Selection of Different Model Parameters

**Figure 1:**
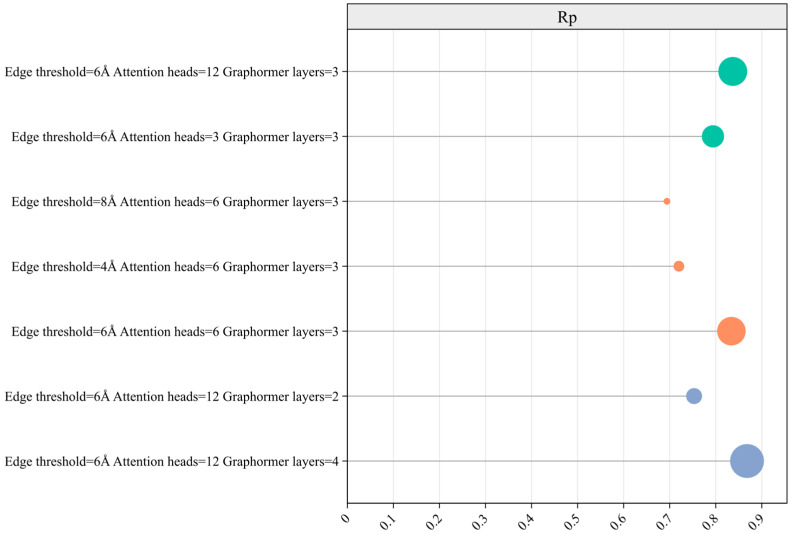
*R_p_* of different model parameters. The horizontal axis represents the *R_p_* value of different parameter groups. The vertical axis represents the value of each parameter. Adjusted parameter groups of the same parameter type are represented by circles of the same color.

The model incorporates three key parameters: edge threshold, number of attention heads, and number of Graphormer layers. The edge threshold defines the maximum distance at which water molecules are considered to form bonds with proteins or ligands. When the distance between a water molecule and a protein or ligand is less than the edge threshold, an edge is generated between them. The attention heads parameter controls the number of heads in the multi-head attention mechanism, and Graphormer layers parameter defines the number of layers used in Graphormer model. Based on prior experience, the edge threshold is adjusted within the range of 4 Å, 6 Å, and 8 Å; the attention heads within 3, 6, and 12; and the Graphormer layers within 2, 3, and 4. This results in a total of 7 parameter combinations. As shown in Table 1 and Figure 1, the model achieves the highest performance (*R_p_* = 0.868 and *RMSE* = 1.27) when the edge threshold is 6 Å, the number of attention heads is 12, and the number of Graphormer layers is 4.

From Table 1, it can be concluded that among the seven parameter groups, the proposed model performs best when the edge threshold for forming bonds between water molecules and proteins or ligands is 6 Å, with six attention heads and four Graphormer layers. At this setting, the *R_p_* reaches 0.868, and the *RMSE* is 1.27. When the edge threshold values are set to 4 Å and 8 Å, the *R_p_* decreases to varying degrees. Therefore, it is suggested that an edge threshold of 6 Å is more effective in capturing interactions between proteins, ligands, and water molecules. However, as the threshold increases, it may introduce more information into the graph structure for subsequent feature extraction, which can also lead to redundancy. After determining the optimal edge threshold, we conducted experiments to assess the impact of the number of attention heads on model performance. The results showed that when the number of attention heads was set to 12, the predictive performance of the model peaked, however, the *R_p_* value of attention heads, of which there were six, only increased by 0.003.

Therefore, it can be inferred that while increasing the number of attention heads enhances the performance of the Graphormer, it may also introduce redundancy into the model. After establishing the optimal settings for edge threshold and attention heads, we examined the model’s performance by varying the number of Graphormer layers. The *R_p_* value consistently increased with the addition of more Graphormer layers; however, this also led to longer training times. To strike a balance between model performance and computational efficiency, we ultimately selected four layers for the Graphormer.

### 2.3. Effects of Introducing Water into the Model

To investigate the influence of water molecules on predicting binding affinity, the proposed method utilized two different approaches to calculate the sites of water molecules in binding pockets, and fused predicted water molecules with complex structures without water molecules, respectively. The complete complex structure, including proteins, ligands and water molecules, was converted into a graph structure for subsequent feature extraction and regression. For the model without the water network, the proposed method used the protein and ligand information from the complex structure gained from the dataset, converting it into a graph structure for subsequent feature extraction and regression. As shown in Table 2, when the model input includes water networks, the *R_p_* is 0.154 higher than that in the case of only proteins and ligands graphs as input. This demonstrates that incorporating a water network into the model’s input graph significantly enhances the prediction of binding affinity. Due to the involvement of water molecules as donors and acceptors in the formation of hydrogen bonds, they can have an impact on the interaction between proteins and ligands. Experimental results indicate that including water molecules in the binding pocket improves the model’s accuracy in predicting protein–ligand binding affinity.

### 2.4. Assessment of GraphWater-Net and State-of-the-Art Methods

**Figure 2:**
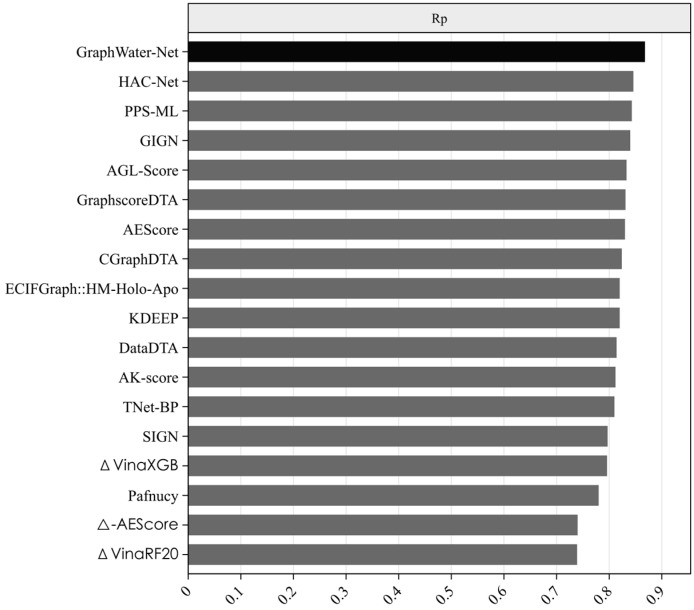
*R_p_* of state-of-the-art methods in grey bars and proposed method in a black bar. The horizontal axis represents the *R_p_* value of different methods. The vertical axis represents the different methods.

**Figure 3:**
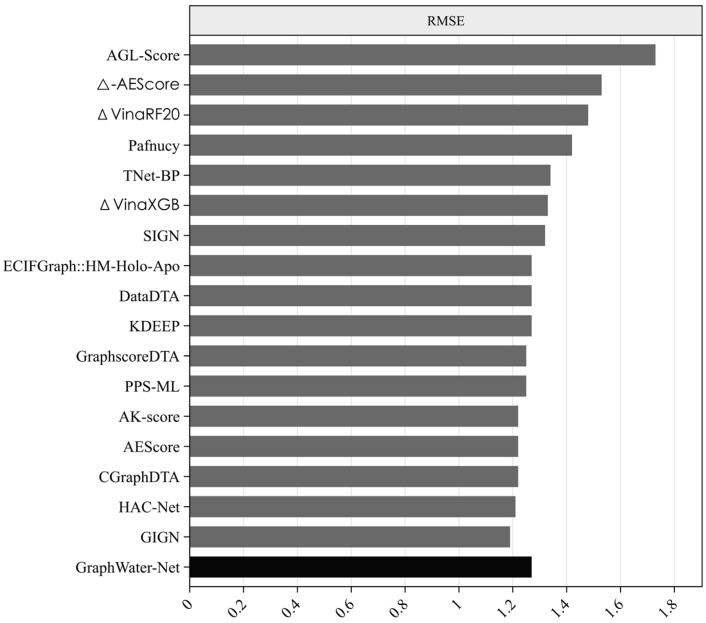
*RMSE* of state-of-the-art methods in grey bars and proposed method in a black bar. The horizontal axis represents the *RMSE* value of different methods. The vertical axis represents the different methods.

Many state-of-the-art methods have been proposed for predicting protein–ligand binding affinity. We selected 17 of these methods, as shown in Table 3, including AEScore [36], DataDTA [37], HAC-Net [38], GraphscoreDTA [39], CGraphDTA [40], PPS-ML [41], SIGN [42], TNet-BP [43], K_DEEP_ [44], Pafnucy [45], and several others. Among them, methods such as ECIFGraph::HM-Holo-Apo, GraphscoreDTA, CGraphDTA, SIGN, and GIGN are GNN-based approaches. For example, GraphscoreDTA uses GNN modules to extract features of proteins, ligands, and pocket–ligand interactions, while CGraphDTA combines multiscale CNN and GNN architectures to represent drug and protein structures. All the methods have been trained on the PDBbind refined set (version 2016) and tested on the CASF-2016 test set. From Figure 2 and Figure 3, it can be seen that the proposed method, GraphWater-Net, which integrates a water network with a GNN, achieved the best performance, with the highest *R_p_* value, outperforming these methods by margins ranging from 0.022 to 0.129. It also achieved the highest correlation between the predicted affinity value obtained from regression prediction and the experimental affinity value, ranking first. Although the *RMSE* value is not the lowest, it is only 0.08 higher than the method with the lowest *RMSE*, GIGN.

*R_p_* measures the linear correlation between the predicted and actual binding affinity values. The structure of GraphWater-Net incorporates critical interaction patterns between proteins, ligands, and water molecules by integrating predicted hydration sites into the graph structure. By incorporating this complex interaction network, the model can capture the general relationships between these components. Meanwhile, *RMSE* quantifies the absolute prediction errors, indicating how far the model’s predictions are from the actual values. Although GraphWater-Net successfully captures the overall trends in protein–ligand interactions—reflected in a high *R_p_*—it may struggle with precision in specific cases. This difficulty can arise in protein–ligand–water systems with complex or unusual interaction patterns that the model cannot fully represent. As a result, while the overall correlation remains strong, some individual samples may exhibit larger absolute errors, resulting in a higher *RMSE*. We mainly consider *R_p_* because it better reflects the model’s ability to capture the key relationships and trends between input features and binding affinity, which is more relevant to the broader goals of this study. Overall, GraphWater-Net remains competitive among state-of-the-art methods in predicting protein–ligand binding affinity.

### 2.5. Ablation Experiments

To better evaluate the performance of different calculation methods for predicting water molecule sites, we conducted two sets of ablation experiments, with training on the PDBbind refined set (version 2016) and testing on CASF2016. The results of these ablation experiments are presented in Table 4.

We designed two sets of ablation experiments for predicting water molecular sites: one that relies solely on HydraMap [51] for water site prediction, referred to as GraphWater-Net_remove 3D-RISM_; and another that uses only 3D-RISM [52], called GraphWater-Net_remove HydraMap_. Firstly, we assessed and compared the performance of HydraMap, which uses knowledge-based statistical potentials to quantify the interactions between water molecules and their surrounding environment. The *R_p_* value of GraphWater-Net_remove 3D-RISM_ decreased by 0.042 compared to GraphWater-Net, while the *RMSE* increased by 0.06. Secondly, we evaluated and compared the performance of 3D-RISM, which is based on statistical mechanics for predicting water molecules binding sites. The *R_p_* value of GraphWater-Net_remove HydraMap_ decreased by 0.204 compared to GraphWater-Net, and the *RMSE* increased by 0.75. These ablation experiments demonstrate the effectiveness of the strategy of combining both calculation methods over relying on a single approach. Notably, the *R_p_* value of GraphWater-Net_remove 3D-RISM_ was 0.162 higher than that of GraphWater-Net_remove HydraMap_, indicating that HydraMap provides a more accurate calculation of water binding sites than 3D-RISM.

### 2.6. Relationship Between Experimental and Predicted Affinity

**Figure 4:**
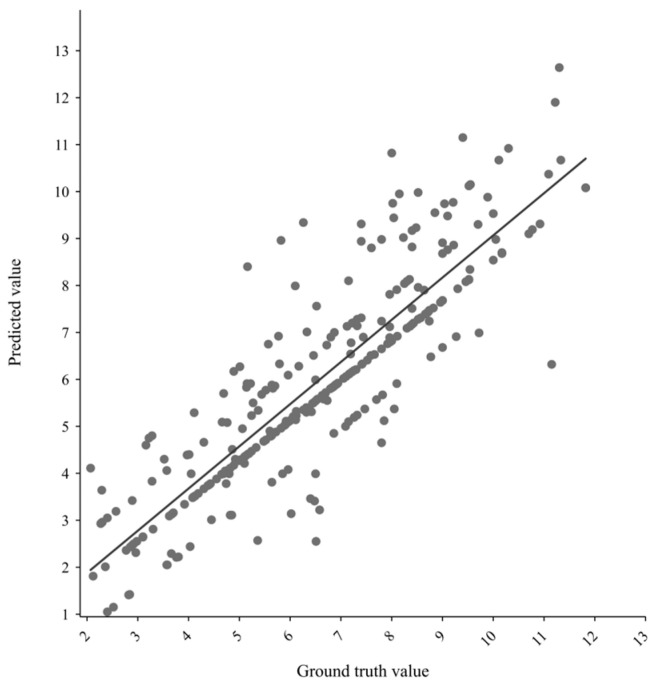
Correlation plot for prediction of GraphWater-Net and ground truth of experiment on CASF2016. The horizontal axis represents the ground truth values of different complexes. The vertical axis represents the predicted values of the different complexes. Each point represents a specific protein-ligand complex.

Figure 4 illustrates that for the 285 complexes in CASF2016, the proposed model’s predictions of binding affinity are generally in close agreement with the ground truth values. Most predicted values cluster around the trend line, indicating strong performance, while only a few exhibit significant deviations from the actual values.

### 2.7. Case Study

**Figure 5:**
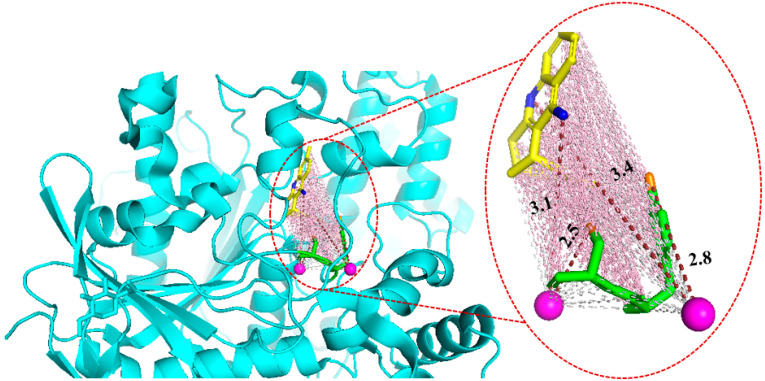
Water-mediated acetylcholinesterase-huprine X interactions in the crystal structure with PDB ID 1E66. Acetylcholinesterase is shown as a diagrammatic representation (cyan), with Tyr121 and Ser122 depicted as sticks (green). The hydroxyl groups of key residues are represented as sticks (orange). Huprine X is also represented as a stick model (yellow), with the amino groups shown as sticks (blue). Water molecules mediating protein–ligand interactions are shown as spheres (purple). Water-mediated interactions in hydrogen bonds are indicated as brown dashed lines with distance metrics (Å). Electrostatic interactions are shown as pink dashed lines, and Van Der Waals forces are shown as grey dashed lines.

The crystal structure with a PDB ID of 1E66 [53] is the complex of acetylcholinesterase with (-)-huprine X, an inhibitor of acetylcholinesterase, which is a pharmacologically significant target in Alzheimer’s treatment [54]. In the crystal structure, Huprine X binds to acetylcholinesterase through a network of water molecules to form critical bridges between the ligand (huprine X) and key residues within the acetylcholinesterase binding pocket, particularly Tyr121 and Ser122 [53]. These water-mediated interactions are crucial for stabilizing the protein–ligand complex, highlighting a binding mechanism that is both indirect and dependent on water molecules, as shown in Figure 5. Water molecules (purple spheres in Figure 5) mediate the interactions between huprine X and the protein acetylcholinesterase by forming hydrogen bond bridges with key residues such as Tyr121 and Ser122 (green), as described in [53]. In the binding pocket, water molecules act as intermediaries, creating hydrogen bonds between the amino group of huprine X and the hydroxyl groups of these residues. Specifically, one water molecule bridges the aromatic hydroxyl group of Tyr121 with the amino group of huprine X, while another water molecule connects the hydroxyl group of Ser122 to neighboring atoms of the ligand. These water-mediated hydrogen bonds enhance the stability of the protein–ligand complex by compensating for structural gaps that would prevent direct bonding between huprine X and the residues. Additionally, these interactions provide flexibility within the binding pocket and preserve the network of hydrogen bonds essential for the integrity of the binding site. While direct hydrogen bonds are generally stronger, water-mediated bonds offer an entropic advantage by stabilizing the solvent environment and lowering the system’s free energy. The X-ray crystallography data of 1E66 confirm the presence of water molecules in strategic positions, bridging huprine X with Tyr121 and Ser122 and reinforcing the binding interaction. These water-mediated interactions play a crucial role in maintaining the stability and functionality of the protein–ligand complex, highlighting the importance of water molecules in modulating the binding process.

However, traditional methods frequently disregard the significance of water in predicting binding affinities, rendering them unable to successfully predict in the aforementioned scenario. Our method, GraphWater-Net, captures the structural and energetic contributions of water molecules by explicitly incorporating water networks into its graph representation of protein–ligand interactions. We illustrate the performance of GraphWater-Net on the 1E66 complex to validate the model’s generalizability to other protein–ligand systems that rely on water-mediated interactions. For this complex, we use other methods to predict its binding affinity and compare the deviation between the predicted and the ground truth values. As shown in Table 5, the proposed method, GraphWater-Net, achieved the best prediction accuracy, with a deviation from the ground truth value of only −0.01. The key difference between GraphWater-Net and other methods is its integration of water networks into the prediction process, which other models lack. The results in Table 5 confirm that GraphWater-Net outperforms models that do not consider water, especially for the 1E66 complex, highlighting its potential in drug discovery.

**Figure 6:**
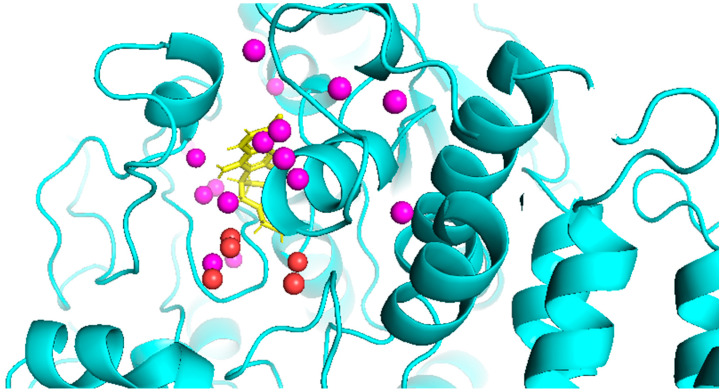
Comparison of predicted and experimental locations of water molecules near or within the ligand-binding site in the 1E66 structure. Water molecules are represented by spheres, with experimental and correctly predicted water molecules shown as purple spheres, and incorrectly predicted water molecules as red spheres. The protein is displayed diagrammatically in cyan, and the ligand is represented a as a yellow stick.

Furthermore, we investigate the stability of the GraphWater-Net model in the presence of perturbations caused by misplaced water molecules within the predicted water network, using the 1E66 protein–ligand complex as an illustrative example. To gain insights into how misplaced water molecules impact the precision of binding affinity predictions, we conducted three comparative experiments for the 1E66 complex, as shown in Table 6. Firstly, we introduced the GraphWater-Net_remove 3D-RISM_ approach, which exclusively employs water molecules forecasted by HydraMap. Secondly, we presented the GraphWater-Net_remove HydraMap_, which solely relies on water molecules predicted by 3D-RISM. Lastly, we presented the GraphWater-Net method in this paper, which integrates water molecules predicted by both 3D-RISM and HydraMap. GraphWater-Net_remove 3D-RISM_ misplaces seven water molecules and has a deviation of 0.2 from the ground truth value. GraphWater-Net_remove HydraMap_ inaccurately places nine water molecules and shows a deviation of 0.28 from the true value. However, by combining both 3D-RISM and HydraMap, GraphWater-Net achieved an excellent prediction with a deviation of −0.01 from the actual binding affinity with only five misplaced water molecules. In Figure 6, we represent the incorrectly predicted water molecules as red spheres, while the correctly predicted ones are represented as purple spheres. Among these, there are 21 water molecules in total near or within the ligand-binding sites of complex 1E66. The GraphWater-Net model demonstrates high stability and accuracy, even when facing perturbations in water molecule placement. The inclusion of both 3D-RISM and HydraMap predictions is highly beneficial because they provide complementary information. This combination significantly reduces prediction errors and ensures that the model can reliably predict binding affinity, even in scenarios where minor errors in water network prediction exist.

## 3. Materials and Methods

### 3.1. Dataset and Assessment Metrics

Accurate experimental measurements are essential for predicting the binding affinity of protein–ligand complexes using computational methods. The PDBbind dataset compiles binding affinity data (*K_d_*, *K_i_*, or *IC*_50_) from experimental measurements linked to biomolecular complex structures in the PDB [55]. Numerous binding affinity prediction studies have leveraged the PDBbind dataset for training and testing (Zhu et al. [56], Wang et al. [57], Sriramulu et al. [58], Soni et al. [59]), making it a widely recognized benchmark. The dataset undergoes stringent screening to ensure the quality and accuracy of both structures and binding data, resulting in a refined set. The PDBbind refined set (version 2016) contains a total of 4057 complex structures, offering a valuable, quality-controlled dataset of protein–ligand complexes, and has become the primary test set in the CASF benchmark [21]. Specifically, the PDBbind core set (version 2016) contains 285 complex structures. In this study, we used 285 complex structures from the PDBbind core set (version 2016) as the test set to evaluate the accuracy of the model, while the remaining 3772 non-overlapping complexes from the PDBbind refined set (version 2016) were used for model training.

In this study, two metrics, *R_p_* and *RMSE*, were employed to measure the performance of the prediction model. *R_p_* measures the correlation strength between two variables. Assuming that *X* represents the experimentally measured binding affinity and *Y* represents the binding affinity predicted by the model, then:

(3) $$R_{p}=\rho_{X,Y}=\frac{cov(X,Y)}{\sigma_{X}\sigma_{Y}}=\frac{E[\left(X-EX\right)\left(Y-EY\right)]}{\sigma_{X}\sigma_{Y}}=\frac{E\left(XY\right)-E(X)E(Y)}{\sqrt{E\left(X^{2}\right)-E^{2}(X)}\sqrt{E\left(Y^{2}\right)-E^{2}(Y)}}$$

As |*R_p_*| becomes closer to 1, the stronger the linear correlation between *X* and *Y*. Conversely, the closer the value of *R_p_* is to 0, the weaker the linear correlation between *X* and *Y*. *RMSE* measures the deviation between predicted and true values. Assuming there is a total of *N* protein ligand complexes, then:

(4) $$RMSE=\sqrt{\frac{\sum_{i=1}^{n}{\left(Y_{i}-X_{i}\right)}^{2}}{N}}$$

The smaller the value of *RMSE*, the better the predictive ability of the model, and the smaller the deviation between the predicted value and the true value.

### 3.2. Graph Representation

**Figure 7:**
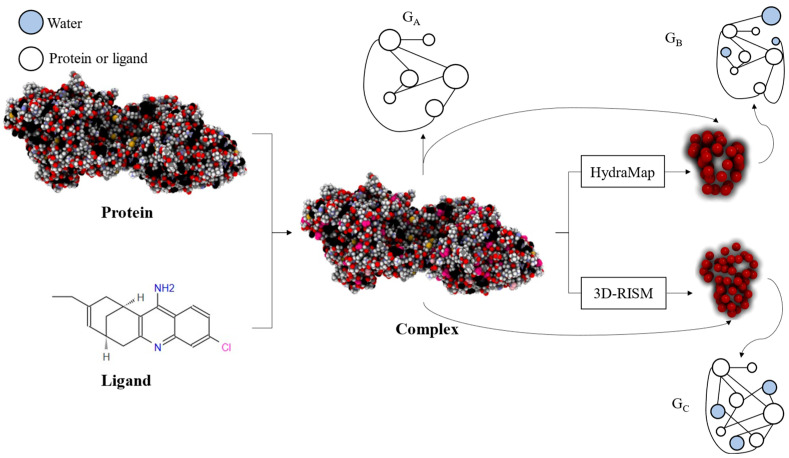
The overall process of graph representation.

The overall workflow is illustrated in Figure 7. Firstly, the protein–ligand complex is represented to obtain *G_A_*. Then, HydraMap and 3D-RISM are used to predict the hydration sites in the binding pocket, resulting in two representations of the complex that incorporate water molecules, referred to as *G_B_* and *G_C_*.

For the generation of *G_A_*, each atom of the protein and ligand in the binding pocket is represented as a node of the graph, and the interactions or covalent bonds between atoms are represented as edges of the graph. The features of nodes and edges are detailed in Table 7 and Table 8, respectively. The node features comprise a total of 58 dimensions. To account for the connectivity between atoms, the protein atom type is defined using ECIF [60]. Edge features primarily focus on the pairwise distance between protein nodes and ligand nodes.

The prediction of the binding sites of water molecules in protein binding pockets can be roughly divided into three popular prediction methods: MD methods, such as WATsite [61]; Monte Carlo methods; and statistical or molecular mechanics-based methods. These methods typically offer fast calculation speeds and high accuracy, such as HydraMap [62] and 3D-RISM [52]. This study utilized HydraMap and 3D-RISM to predict the binding sites of water molecules in the binding pocket, respectively. HydraMap predicts the hydration sites in protein binding pockets based on statistical potential and calculates the desolvation energy. By using the coordinates of ligand molecules, the position of the binding pocket on the protein is identified. A rectangular prism is used to represent this binding pocket, which is divided into grids. A water probe is placed on each grid, and the probability of observing the water probe on each grid is calculated to screen for grids with higher probability. Finally, the mean shift algorithm is used to cluster them, and each cluster center point represents a binding site for water molecules. In this study, HydraMap v1.0 software was used to process the PDB format file of proteins and the Mol2 format file of ligands for analysis of hydration sites. The parameter settings of the software are shown in Table 9. 3D-RISM calculates the equilibrium density distribution of the explicit solvent model around the solute on a 3D grid using the Ornstein Zernike integral equation. Firstly, the rism1d cSPCE-NaCl > cSPCE-NaCl.out command in AmberTools19 is used to perform the calculation of 1D-RISM, obtaining the bulk solvent site susceptibility in the reciprocal space, $\chi^{VV}$. We then use the rism3dsnglpnt command to perform 3D-RISM calculations.

After obtaining the distribution of water molecules in protein binding pockets, a graph representation is generated to simulate the interactions among water molecules, proteins, and ligands. A distance threshold *T* is established. If the distance between water molecules and either proteins or ligands is less than *T*, an edge is added between the corresponding atoms. Consequently, the edge features of *G_B_* and *G_C_* shown in Table 10 primarily focus on the distance between protein nodes, ligand nodes, and water molecule nodes.

### 3.3. Model Construction

**Figure 8:**
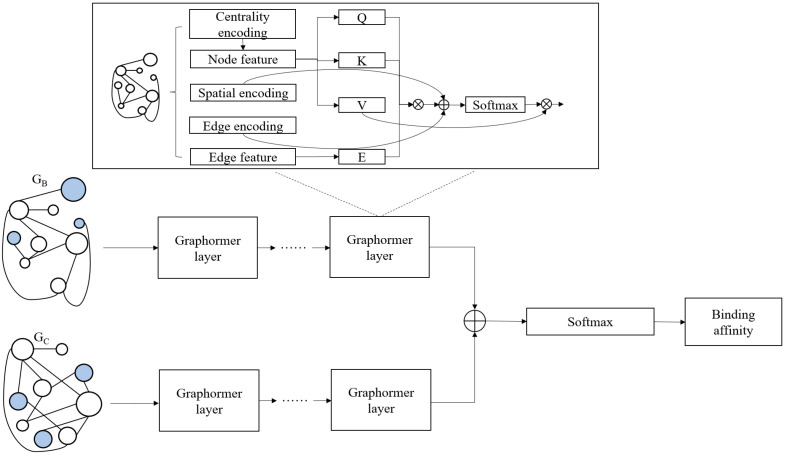
The structure of the proposed model.

This study proposes a model for predicting protein–ligand binding affinity, with the overall architecture of the model illustrated in Figure 8.

Taking the graphs *G_B_* and *G_C_* of the complex introduced into the water network as inputs:

(5) $$G_{B}=\left(V_{B},E_{B}\right),\;V=\left\{v_{0}^{B},v_{1}^{B},\;\cdots ,\;v_{n}^{B}\right\}$$

(6) $$G_{C}=\left(V_{C},E_{C}\right),\;V=\left\{v_{0}^{C},v_{1}^{C},\;\cdots ,\;v_{n}^{C}\right\}$$

Graphormer layers were employed for feature extraction separately. Graphormer, introduced by Ying et al. in 2021, is a variant of the Transformer architecture [34]. The most critical part of Transformer is the self-attention module, and the calculation formula for the attention coefficient is as follows:

(7) $$Attention=Softmax\left(\frac{{(x}_{l}W_{Q})(x_{l}{W_{K})}^{T}}{\sqrt{d}}\right)x_{l}W_{V}$$

In Equation (7), $x_{l}$ represents the *l*-th hidden layer in the network, while $W_{Q},\;W_{K}$ and $W_{V}$ are the three linear transformation matrices used to calculate the query matrix, key matrix, and value matrix, respectively. The variable *d* denotes the dimension of the network.

Graphormer enhances the model’s expressive power through three encoding methods. Firstly, centrality encoding calculates the out degree and in degree of each node. The fundamental idea is to incorporate the centrality of a node as an additional feature alongside its original attributes. This approach not only enriches the node representation but also quantifies the importance of each node in the graph. Next, spatial encoding integrates positional information into node representation, enabling the GNN to better understand spatial relationships among nodes. Here, the shortest path distance between nodes $v_{i}$ and $v_{j}$ serves as the spatial encoding to capture the spatial dependencies of the graph. Finally, edge encoding captures the structural features of edges by calculating edge features associated with the shortest path between $v_{i}$ and $v_{j}$, as well as embedding representations of that path. The edge feature information is integrated into the representation of GNN to better utilize the edge information to enhance the performance of the model. We introduce spatial encoding and edge encoding as bias terms in the attention coefficient:

(8) $$Attention=Softmax\left(\frac{(x_{l}W_{Q}){\left(x_{l}W_{K}\right)}^{T}}{\sqrt{d}}+D(v_{i},v_{j})+\frac{\sum_{i=0,j=0}^{i,j}e_{ij}{\left(W_{ij}^{E}\right)}^{T}}{N}\right)x_{l}W_{V}$$

In Equation (8), $e_{ij}$ represents the feature of the edge connecting node *i* to node *j*, *N* denotes the total number of edges in the graph, and $W_{ij}^{E}$ is the embedding of the shortest path between node *i* and node *j*.

After extracting from *G_B_* and *G_C_* separately using Graphormer, the extracted features are concatenated and input into the Softmax function for regression prediction on affinity values.

## 4. Conclusions

To accurately predict the binding affinity between proteins and ligands, this study proposed a method based on the Graphormer architecture. Firstly, we employed two distinct methods for predicting water molecule sites to identify hydration sites in the binding pockets of each complex in the PDBbind refined set (version 2016). These predicted water sites were integrated with the complex structures, which initially contained only protein and ligand molecules, transforming the data into a graph representation where atoms serve as nodes and bonds, and interactions act as edges. The resulting graph structures were then separately fed into the Graphormer network for extracting edge and node features, followed by regression predictions for binding affinity values. To assess the impact of water molecules on predicting binding affinity, we also included a complex graph without the water network in the feature extraction process. The experimental results demonstrated that the inclusion of water molecules enhances the accuracy of predicting protein–ligand binding affinity, and the proposed model performed well on the CASF-2016 benchmark. However, this study has not yet explored whether the inclusion of water molecules affects the position of the original receptor and ligand molecules in the complex. Further research is needed to investigate the influence of water networks on structural changes within the complex, as well as to enhance the model by conducting a more detailed analysis of hydrophilic and hydrophobic interactions. Additionally, we aim to investigate the dynamic behavior of binding pockets through molecular dynamics simulations or flexibility analysis, providing additional insights into their flexibility and conformational changes.

## Figures and Tables

**Table 1:** *R_p_* and Root Mean Square Error (*RMSE*) of seven models with different parameters, including edge threshold, attention heads and Graphormer layers.

	Parameters			*R_p_*	*RMSE*
	Edge Threshold	Attention Heads	Graphormer Layers		
1	6 Å	6	3	0.834	1.330
2	4 Å	6	3	0.720	1.570
3	8 Å	6	3	0.694	1.610
4	6 Å	3	3	0.794	1.430
5	6 Å	12	3	0.837	1.370
6	6 Å	12	4	0.868	1.270
7	6 Å	12	2	0.753	1.500

**Table 2:** *R_p_* and *RMSE* of complex binding affinity with and without water network.

	*R_p_*	*RMSE*
With water network	0.868	1.27
Without water network	0.714	1.61

**Table 3:** *R_p_* and *RMSE* of state-of-the-art methods and proposed method.

	Method	*R_p_*	*RMSE*
RF-based	ΔVinaRF20 [46]	0.739	1.48
XGBoost-based	ΔVinaXGB [47]	0.796	1.33
NN-based	Δ-AEScore [36]	0.740	1.53
	AEScore [36]	0.830	1.22
CNN-based	AK-score [48]	0.812	1.22
	DataDTA [37]	0.814	1.27
	HAC-Net [38]	0.846	1.21
	TNet-BP [43]	0.810	1.34
	K_DEEP_ [44]	0.820	1.27
	Pafnucy [45]	0.780	1.42
GBT-based	PPS-ML [41]	0.843	1.25
	AGL-Score [49]	0.833	1.73
GNN-based	ECIFGraph:HM-Holo-Apo [5]	0.820	1.27
	GraphscoreDTA [39]	0.831	1.25
	CGraphDTA [40]	0.824	1.22
	SIGN [42]	0.797	1.32
	GIGN [50]	0.840	1.19
	GraphWater-Net	0.868	1.27

**Table 4:** *R_p_* and *RMSE* of complex binding affinity with and without 3D-RISM and HydraMap.

Model	*R_p_*	*RMSE*
GraphWater-Net	0.868	1.27
GraphWater-Net_remove 3D-RISM_	0.809	1.28
GraphWater-Net_remove HydraMap_	0.647	2.01

**Table 5:** Prediction value, ground truth value and prediction deviation of binding affinity of complex 1E66 using different predicted methods.

Model	Ground Truth Value	Prediction Value	Prediction Deviation
ΔVinaRF20	9.89	11.71	+1.82
ΔVinaXGB		7.46	−2.43
Δ-AEScore		7.51	−2.38
AK-score		11.63	+1.74
AEScore		8.27	−1.62
DataDTA		10.65	+0.76
HAC-Net		11.01	+1.12
GraphscoreDTA		10.25	+0.36
CGraphDTA		9.93	+0.04
PPS-ML		9.94	+0.05
SIGN		7.99	−1.90
GIGN		9.86	−0.03
TNet-BP		9.17	0.72
K_DEEP_		9.54	−0.35
AGL-Score		9.77	−0.12
Pafnucy		12.01	+2.12
GraphWater-Net		9.88	−0.01

**Table 6:** Comparison of binding affinity prediction for the 1E66 complex using different water molecule prediction models with and without 3D-RISM and HydraMap.

Model	Binding Affinity	Prediction Deviation
GraphWater-Net	9.88	−0.01
GraphWater-Net_remove 3D-RISM_	9.69	−0.20
GraphWater-Net_remove HydraMap_	10.17	0.28

**Table 7:** Node features in *G_A_*.

Node Features	Numbers	Content
Residue type	20	VAL, ILE, LEU, GLU, GLN, ASP, ASN, HIS, TRP, PHE, TYR, ARG, LYS, SER, THR, MET, ALA, GLY, PRO, CYS
Protein atom type	22	C,4,2,2,0,0 N,4,1,2,0,0 S,2,2,0,0,0 C,4,3,0,1,1 N,3,1,2,0,0 C,4,3,0,0,0 C,4,1,3,0,0 N,3,2,1,1,1 S,2,1,1,0,0 C,4,3,1,0,1 N,3,2,0,1,1 C,4,3,1,0,0 O,2,1,1,0,0 N,4,2,1,0,0 N,3,2,1,0,0 N,4,1,3,0,0 C,6,3,0,0,0 C,4,2,2,0,1 C,4,2,1,1,1 O,2,1,0,0,0 N,3,3,0,0,1 C,5,3,0,0,0
Ligand atom type	9	C, N, O, S, F, Cl, Br, I, P
Coordinates	1	*x*, *y*, *z*
Implicit valence electrons	1	Any positive integer
Aromatic atom (Yes/No)	2	1, 0
Ring atom (Yes/No)	2	1, 0
Number of H atoms	1	Any positive integer

**Table 8:** Edge features in *G_A_*.

Edge Features	Numbers	Level	Cutoff
Ligand–ligand distance	1	Heavy atom	6 Å
Protein–ligand distance	1	*C_α_* atom, heavy atom	
Protein–protein distance	1	*C_α_* atom	

**Table 9:** Parameters for HydraMap v1.0.

Parameter	Description	Value
D	The distance between the cubic boundary and the nearest ligand atom	4.0 Å
Cutoff	Definition favorable PMF values in water site clustering	5.0 Å
radius	The distance defining the region around the center point	2.0 Å
N	The least number of grids in a cluster for a valid hydration site	1.0 Å
distance	A value defining the distance between the oxygen atom of the water molecule and the protein/ligand atom	4.0 Å

**Table 10:** Edge features in *G_B_* and *G_C_*.

Edge Features	Numbers	Level	Cutoff
Ligand–ligand distance	1	Heavy atom	6 Å
Protein–ligand distance	1	*C_α_* atom, heavy atom	
Protein–protein distance	1	*C_α_* atom	
Water–ligand distance	1	Oxygen atom, heavy atom	
Water–protein distance	1	Oxygen atom, *C_α_* atom	
Water–water distance	1	Oxygen atom	

## Data Availability

Code and data for GraphWater-Net and other baselines are available at https://github.com/guofei-tju/GraphWater-Net accessed on 10 March 2024.
